# Novel Therapeutic Targets for the Treatment of Right Ventricular Remodeling: Insights from the Pulmonary Artery Banding Model

**DOI:** 10.3390/ijerph18168297

**Published:** 2021-08-05

**Authors:** Argen Mamazhakypov, Natascha Sommer, Birgit Assmus, Khodr Tello, Ralph Theo Schermuly, Djuro Kosanovic, Akpay Sh. Sarybaev, Norbert Weissmann, Oleg Pak

**Affiliations:** 1 Excellence Cluster Cardio-Pulmonary Institute (CPI), Universities of Giessen and Marburg Lung Center (UGMLC), Member of the German Center for Lung Research (DZL), Justus-Liebig University, 35392 Giessen, Germany; Argen.Mamazhakypov@innere.med.uni-giessen.de (A.M.); Natascha.Sommer@innere.med.uni-giessen.de (N.S.); Birgit.Assmus@innere.med.uni-giessen.de (B.A.); Khodr.Tello@innere.med.uni-giessen.de (K.T.); Ralph.Schermuly@innere.med.uni-giessen.de (R.T.S.); djurokos13@gmail.com (D.K.); oleg.pak@innere.med.uni-giessen.de (O.P.); 2 Department of Cardiology, Justus-Liebig University, 35390 Giessen, Germany; 3 Department of Pulmonology, I.M. Sechenov First Moscow State Medical University (Sechenov University), 119992 Moscow, Russia; 4 Department of Mountain and Sleep Medicine and Pulmonary Hypertension, National Center of Cardiology and Internal Medicine, Bishkek 720040, Kyrgyzstan; ak_sar777@mail.ru

**Keywords:** pulmonary hypertension, right ventricular failure, pulmonary artery banding

## Abstract

Right ventricular (RV) function is the main determinant of the outcome of patients with pulmonary hypertension (PH). RV dysfunction develops gradually and worsens progressively over the course of PH, resulting in RV failure and premature death. Currently, approved therapies for the treatment of left ventricular failure are not established for the RV. Furthermore, the direct effects of specific vasoactive drugs for treatment of pulmonary arterial hypertension (PAH, Group 1 of PH) on RV are not fully investigated. Pulmonary artery banding (PAB) allows to study the pathogenesis of RV failure solely, thereby testing potential therapies independently of pulmonary vascular changes. This review aims to discuss recent studies of the mechanisms of RV remodeling and RV-directed therapies based on the PAB model.

## 1. Introduction

Right ventricular (RV) remodeling is characterized by complex rearrangements of the myocardial micro- and macrostructure, resulting from increased accumulation of extracellular matrix in the myocardium, capillary rarefaction, inflammatory/immune cell infiltration, and cardiomyocyte hypertrophy in response to pressure or volume overload, which further leads to adverse alterations in the myocardial performance, eventually resulting in right heart failure and premature death [1]. RV remodeling develops in the various forms of pulmonary hypertension (PH), which is defined by a mean pulmonary artery pressure (mPAP) of more than or equal to 20 mm Hg and a pulmonary vascular resistance (PVR) ≥3 Wood units (WU) for pre-capillary forms of PH measured by right heart catheterization [2,3]. PH combines heterogeneous pulmonary vascular conditions, classified into five groups as follows: Group 1—pulmonary arterial hypertension (PAH), including idiopathic, heritable, and drug/toxin-induced PH; Group 2—PH due to left heart disease; Group 3—PH due to lung disease and/or chronic hypoxia; Group 4—PH due to chronic thromboembolism; Group 5—PH with unclear multifactorial mechanisms. In patients with PH, RV afterload in most cases increases gradually over years, which provides enough time for the manifestation of adaptive mechanisms to maintain RV function [4,5]. RV wall thickening (RV hypertrophy), as one of the early mechanisms of the RV adaptation to pressure overload, develops in the initial (adaptive) stage of RV remodeling, and serves to generate higher pressure to overcome increased afterload. The initial changes in the RV structure upon gradual pressure overload aim to maintain RV function against the increased RV afterload at the expense of structural changes, including cardiomyocyte hypertrophy and moderate fibrosis (adaptive RV remodeling). However, at some point over the course of the disease, the RV loses its ability to withstand against sustained pressure overload due to inadequate or excessive myocardial fibrosis, impaired myocardial capillarization, and cardiomyocyte apoptosis, defining the transition to maladaptive RV remodeling, which ultimately results in RV failure.

The currently approved therapeutics for the treatment of PAH (idiopathic and heritable PAH) can improve RV function along with the improvement of pulmonary hemodynamics. However, it remains challenging to differentiate RV-specific effects from the pulmonary vascular effects. Under the majority of those treatments, the improvement of RV function closely correlates with the degree of RV afterload reduction, suggesting complete afterload-dependent effects. In afterload-dependent animal models of RV remodeling, reduction of PAP and PVR to normal values completely reverses RV remodeling to the healthy state. Similarly, in PAH patients, after a drastic decrease in PVR, a complete reversal of RV remodeling is observed, and such cases are seen in PAH patients after lung transplantation [6], after left ventricular assist devices (LVAD) placement [7], and in CTEPH patients after successful pulmonary endarterectomy [8]. Although, a report suggests that in PH due to lung diseases, RV function cannot be restored completely [9]. Thus, there are two pharmacological avenues to exploit in the therapy of RV failure: (1) agents targeting the pulmonary vasculature with profound pulmonary vascular reverse remodeling effects with subsequent RV afterload reduction and reversal of RV remodeling, and (2) therapies targeting the RV directly to improve its adaptation to pressure overload.

Simple use of standard drugs for therapy of left ventricular (LV) failure in RV failure has not shown positive results. For example, inhibitors of the renin–angiotensin–aldosterone system (RAAS) can improve RV function in congenital heart disease-associated RV failure [10]. However, the clinical studies on angiotensin-converting enzyme (ACE) inhibitors in PAH demonstrated controversial results and therefore, no consensus opinion on the use of ACE inhibitors in patients with PAH exists [11]. In line with this, the clinical trials on β-blockers demonstrated negative or no clear beneficial effects in patients with PAH [12].

Unfortunately, currently approved PAH therapies have limited potential to reduce afterload, despite overall functional improvements. For example, a meta-analysis has revealed that PAH-specific therapies decreased mPAP only by 2.87 mmHg during 14.3 weeks of treatment, suggesting that they have little effect on PAP decrease [13]. In addition, another meta-analysis has demonstrated that a 12-week treatment with currently available PAH therapies had no favorable direct effect on right heart function [14]. However, recent studies suggest that upfront dual- and triple-combination therapies improve RV function in patients with PAH [15,16,17], although these beneficial effects of combination therapies were likely due to lowered PAP and PVR, and not directly acting on the RV [18].

In summary, it remains challenging to develop RV-directed therapies in PH patients due to limited knowledge of the pathobiology of RV hypertrophy and failure. In this review, we summarize potential RV-directed pharmacological agents that are in preclinical studies using the experimental pulmonary artery banding (PAB) model, which targets various cellular processes such as myocardial fibrosis, metabolic dysfunction, myocardial inflammation, regenerative potential, and the challenges and opportunities in developing such treatments.

## 2. Pulmonary Artery Banding as a Model in Right Ventricular-Directed Drug Discovery

**Figure 1:**
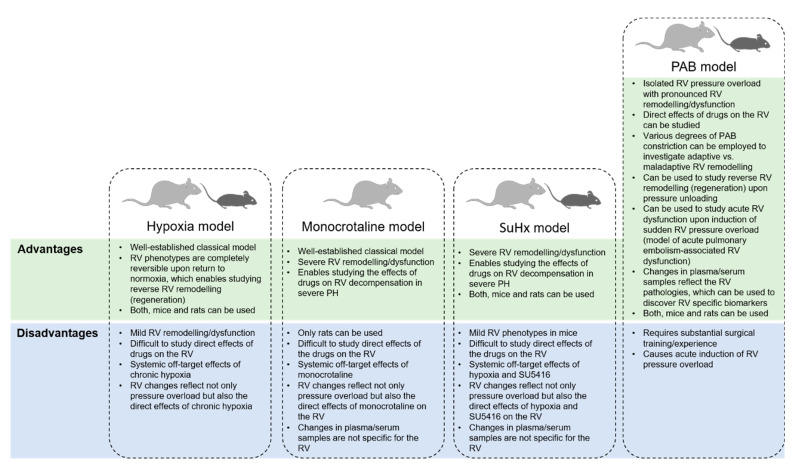
Advantages and disadvantages of pulmonary artery banding (PAB) model of right ventricular (RV) failure.

Understanding the processes underlying a disease can be achieved by using a solid animal model for experimental studies. The ideal animal model is expected to mimic the human disease processes and to be easily reproducible. Although several very well characterized animal models exist for LV failure, only the PAB model can so far be used reliably for the studies of RV failure. Pressure overload is the most common etiology for RV hypertrophy and failure observed in patients with PAH, and PH associated with other diseases. The PAB model of RV hypertrophy and failure recently has gained increased attention as one of the crucial tools to study the mechanisms underlying RV remodeling and evaluate potential RV-directed treatment options. The most important advantage of this model compared to classical animal PH models (chronic hypoxia-induced PH and monocrotaline (MCT)-induced PH) and to the distinctive SuHx-PH model (combination of a vascular endothelial growth factor receptor (VEGFR) antagonist, Sugen 5416 (Su5416), and exposure to 3 weeks of chronic hypoxia in rats) is that it allows a detailed and specific exploration of the mechanisms of RV dysfunction and remodeling independent of pulmonary vascular changes (Figure 1). Creating fixed RV pressure overload by placing a metal clip on the main pulmonary artery or its ligation with a suture to decrease the main pulmonary cross-sectional area to around 70% is a basic feature of the PAB model, and it is used in various animals, including small (mice and rats) and large animals such as sheep, pigs, and lambs.

PAB in mice and rats is the most reliable model of RV hypertrophy and failure, and has several advantages over other animal models (Figure 1). For example, mouse PAB model allow to discover RV-specific drug targets by exploiting gene-specific loss-of-function or gain-of-function mice in specific cell types at specific time points, as well as to test the effect of potential pharmacological agents on RV function and structure in an easy, fast, cost-effective, and reproducible manner. In addition, using the same strain of animals helps to exclude heterogeneity of the results due to genetic variations. This allows for experimental designs to evaluate specific molecular mechanisms in smaller animal numbers. It is essential to recognize that, in the PAB model, the degree of long-term RV remodeling and RV failure is directly proportional to the initial degree of PAB [19]. Therefore, it is necessary to keep equivalence of PA ligation size between groups when comparing subsequent remodeling responses in different groups of animals. Importantly, small animal PAB models can be used also to test potential cardiotoxic effects of the drugs developed for other diseases as well as in PH before moving forward to clinic trials [20]. It was shown in isolated perfused rat hearts that bosentan decreased RV contractility in a dose-dependent manner in the hypertrophied RV but not in the normal RV [21]. Earlier studies demonstrated that PAB rats display compensated RV hypertrophy without signs of RV failure compared to SuHx or MCT-challenged rats, which led authors to conclude that the PAB model cannot fully recapitulate human RV failure observed in PAH patients [22]. However, the RV phenotypes observed in PAB models are causally related to the degree of pressure overload, as tighter PAB constriction leads to decompensated RV failure with high mortality [19]. Interestingly, a recent study has demonstrated that the rat PAB model develops all hemodynamic and histological features of RV failure to a similar degree as found in MCT and SuHx rats [23].

**Figure 2:**
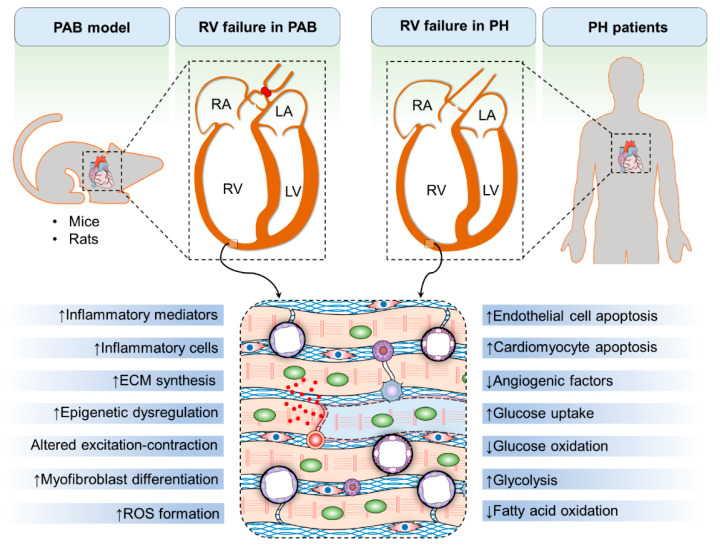
The pulmonary artery banding (PAB) model of right ventricular (RV) failure recapitulates many features of RV failure observed in pulmonary hypertension (PH) patients. PAB in mice and rats causes RV dilatation and RV wall hypertrophy similar to those observed in PH patients. Many of the pathological characteristics of RV remodeling in PH patients can be seen in the RV of PAB rodents, including increased myocardial inflammation, upregulation of inflammatory mediators, accumulation of extracellular matrix (ECM), myofibroblast differentiation, alterations of epigenetic processes, dysregulation of cardiomyocyte excitation–contraction coupling, oxidative stress, apoptosis of cardiomyocytes and endothelial cells, downregulation of proangiogenic factors, increased glucose uptake and glycolysis, and decreased glucose and fatty acid oxidation.

Collectively, small animal PAB models can be used to define hemodynamic, mechanical, neurohormonal, cellular, and molecular changes during RV hypertrophy and failure and to evaluate the potential efficacy of novel therapeutics (Figure 2). Therefore, more widespread exploitation of this model can help to develop therapies focusing on RV function in cardiovascular diseases associated with RV failure and ultimately may allow developing strategies to improve symptoms, quality of life, hemodynamics, and survival of patients.

## 3. Mechanisms of Right Ventricular Hypertrophy and Failure

Despite extensive research, the cellular signaling pathways underlying the development of RV hypertrophy and its transition to RV failure are not fully understood. The pathobiology of RV remodeling is characterized by development and progression of several pathological processes, including augmented myocardial fibrosis [24], inflammation [25], impaired myocardial capillarization [26], dysregulated neurohormonal homeostasis [27], altered metabolism [28], mitochondrial dysfunction [29], and increased production of reactive oxygen species (ROS) [30]. However, cellular mechanisms that regulate and govern such processes in pressure overload-induced RV remodeling remain incompletely understood. Nevertheless, recent studies have identified several dysregulated signaling pathways that drive such pathological processes in RV remodeling in response to pressure overload. Each pathological process has been shown to be regulated by distinct signaling pathways. For example, tyrosine kinase and mitogen-activated protein kinase (MAPK) signaling pathways have been shown to contribute to cardiac fibroblast transdifferentiation and extracellular matrix (ECM) synthesis resulting in adverse myocardial fibrosis [31], while cardiomyocyte loss and capillary rarefaction were results of augmented activation of apoptotic signaling pathways. Myocardial inflammation was associated with toll-like receptor 9 (TLR9)–nuclear factor kappa-light-chain-enhancer of activated B cells (NF-kB) signaling pathway activation in myocardial macrophages [32]. Similarly, activation of mast cells, which release a plethora of inflammatory mediators, has also been shown to worsen RV remodeling [32]. In addition, increased oxidative stress was also found to contribute to adverse RV remodeling in response to pressure overload [33]. Similarly, metabolic alterations such as altered glucose and fatty acid oxidation (FAO) were also demonstrated to contribute to the development of RV failure [34]. Finally, epigenetic alterations such as increased histone deacetylase (HDAC) and bromodomain (BRD) activities were also found to contribute to RV functional decline [35]. Taken together, all these pathways have emerged as promising targets to protect the pressure-overloaded RV in animal models of PH.

## 4. Pharmacotherapies Modulating Myocardial Fibrosis

Myocardial fibrosis is characterized by altered architecture and composition of ECM due to fibroblast proliferation and their transdifferentiation into myofibroblasts, which eventually leads to impaired cardiomyocyte contractility and cardiac dysfunction [36]. Continuous exposure to various profibrotic factors such as pressure overload drives excessive ECM deposition, resulting in impaired tissue function and heart failure. In various LV pathologies, the extent and severity of myocardial fibrosis are associated with adverse outcome [37]. Similarly, excessive fibrosis of the RV in patients with PH is associated with more pronounced hemodynamic alterations and poor outcome [24,36]. Despite the clinical importance of myocardial fibrosis in both LV and RV pathologies, the mechanisms underlying myocardial fibrosis development and progression are not completely understood and there are no approved therapies targeting this process [24,36]. However, recent studies have identified some dysregulated signaling pathways contributing to myocardial fibrosis, which have subsequently been targeted to attenuate myocardial fibrosis in PAB models (Figure 2).

### 4.1. Modulators of Mitogen-Activated Protein Kinases

**Figure 3:**
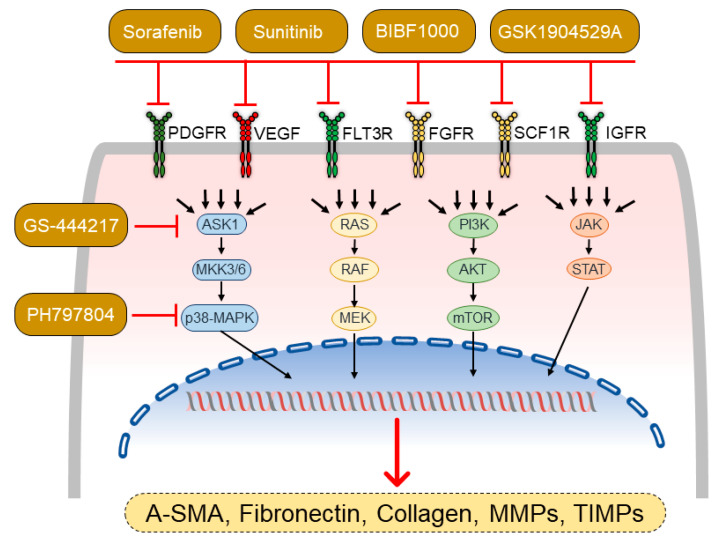
Mechanisms of agents identified to attenuate myocardial fibrosis in pulmonary artery banding (PAB)-induced right ventricular (RV) remodeling. BIBF1000, a nintedanib analogue that targets VEGFRs, PDGFRs, and FGFRs, GSK1904529A, a specific IGF-1R inhibitor, sorafenib, a multi-kinase inhibitor of PDGFRs, VEGFRs, Flt3, c-Kit, c-RAF, and b-RAF, and sunitinib, an inhibitor of PDGFRs, VEGFRs, Flt3, KIT, CSF1R, and RET have been shown to attenuate myocardial fibrosis by inhibiting the activity of cardiac fibroblasts and attenuating the expression of profibrotic genes such as α-SMA (actin alpha 2), fibronectin, collagen, MMPs (matrix metalloproteinases), and TIMPs (tissue inhibitors of metalloproteinases), thus improving RV function in animal models of PAB.

Dysregulation of MAPKs, including extracellular signal-regulated kinases (ERK1 and 2), c-jun N-terminal protein kinases (JNK), and p38 kinases, has been shown to contribute to myocardial fibrosis and adverse LV remodeling in response to pressure overload [38,39]. Various extracellular and intracellular stimuli such as growth factors, phorbol esters, and oxidative stress are known to activate MAPKs, which subsequently translocate from the cytoplasm to the nucleus and regulate the activity of various transcriptional factors [38]. Like in LV failure, dysregulation of various MAPKs has been shown to be involved in the development of pressure overload-induced adverse RV remodeling. For example, apoptosis signal-regulating kinase 1 (ASK1), a ubiquitously expressed MAP3K (mitogen-activated protein kinase kinase), is activated in the RV tissue in response to pressure overload, and administration of the specific ASK1 inhibitor GS-444217, starting 1 week after PAB surgery when RV remodeling is established, improves RV function and decreases RV fibrosis [40]. Additionally, ASK1 inhibition prevents the progressive increase in RV dilation induced by PAB and is associated with substantial increase in cardiac output [40]. Cardiac fibroblasts have been shown to be the main cell type displaying increased ASK1 pathway activation in RV remodeling, and treatment of RV cardiac fibroblasts with GS-444217 dose-dependently reduces cardiac fibroblast migration and collagen secretion in response to transforming growth factor beta (TGF-β) stimulation [40]. One of the downstream mediators of ASK1, p38 MAPK activity, is also increased in the RV of PAB mice, and its inhibition with the specific inhibitor PH797804 improves RV function and fibrosis, while maintaining RV hypertrophy [41]. However, a phase II clinical trial with the ASK1 inhibitor selonsertib for 24 weeks did not lead to a significant reduction in PVR or improved RV function in patients with PAH (NCT02234141) [42]. The benefits of p38 MAPK inhibition are due to its ability to prevent (1) cardiac fibroblast transdifferentiation into myofibroblast, (2) extracellular matrix production via attenuation of myocardin-related transcription factor A (MRTF-A) translocation from the cytoplasm to the nucleus, and (3) partly via inhibition of SMAD1/3/8 activation. Taken together, among various MAPKs, ASK1 and p38 MAPK have been shown to contribute to adverse RV remodeling mainly by regulating cardiac fibroblast-mediated ECM synthesis and deposition. Specific inhibition of these MAPKs substantially improves RV function and remodeling in the mouse PAB model (Figure 3).

### 4.2. Tyrosine Kinase Inhibitors

Tyrosine kinases are a group of enzymes consisting of around 90 members, which play a crucial role in translating various extracellular stimuli such as growth factors and cytokines into intracellular signaling pathways that govern diverse cellular functions such as growth, differentiation, metabolism, migration, and apoptosis [43]. Tyrosine kinases catalyze the transfer of a phosphate residue from ATP to tyrosine residues in target proteins, ultimately determining functions of the phosphorylated substrate proteins such as activity, subcellular location, and stability. Many of the tyrosine kinases have been found to be dysregulated in various pathologies, including cancer and pulmonary hypertension, which led to the development of tyrosine kinase inhibitors (TKIs) for the treatment of these diseases [44]. However, many of the TKIs have been shown to cause cardiotoxicity in cancer patients [45]. Thus, possible cardiotoxic effects of the TKIs, shown to improve pulmonary vascular remodeling, should be tested preclinically in PAB models to exclude their adverse effects on the RV before transfer into clinic development. Some of the TKIs have already been proven not only to be safe, but also to improve RV function in PAB models, mostly by attenuating myocardial fibrosis. For example, sorafenib and sunitinib, both inhibitors of multiple kinases, including PDGFRs (platelet-derived growth factor receptors), VEGFRs, Flt3 (fms-related receptor tyrosine kinase 3), c-Kit, c-RAF, and b-RAF, and sunitinib, an inhibitor of PDGFRs, VEGFRs, Flt3, KIT, CSF1R (colony-stimulating factor 1 receptor), and RET, have been shown to improve RV function by inhibiting profibrotic processes when administered for 2 weeks in PAB rats with established RV hypertrophy [46]. Moreover, another TKI, BIBF1000, a nintedanib analogue that targets VEGFRs, PDGFRs, and FGFRs (fibroblast growth factor receptor), had no detrimental effects on the RV during 35 days of treatment of PAB rats [47]. In addition, GSK1904529A, a specific insulin-like growth factor 1 receptor (IGF-1R) inhibitor, administered to PAB mice with established RV remodeling could improve RV function and attenuated RV hypertrophy [48], suggesting that IGF-1R signaling may play a role in hypertrophic processes [48]. Since pirfenidone has been shown to improve LV function and fibrosis in response to pressure overload, it has also been studied in PAB models for its therapeutic effects in the RV [49]. However, these studies have revealed that pirfenidone does not improve RV function, in spite of attenuated myocardial fibrosis in PAB mice [50], and neither exerts any antifibrotic effects nor improves RV function in PAB rats [51]. Furthermore, it has been shown that pharmacological inhibition of mammalian target of rapamycin complex 1 (mTORC1), which is a downstream mediator of several growth factors, reversed cardiomyocyte hypertrophy and RV remodeling, improved RV contractility, and prevented development of RV fibrosis [52]. However, data on effects of mTORC1 inhibition on RV failure in PAB model are missing. In summary, these studies revealed that some of the TKIs may have beneficial effects on the RV by attenuating myocardial fibrosis (Figure 3).

## 5. Pharmacotherapies Modulating Metabolic Dysregulation

In the healthy myocardium, 95% of the ATP is derived from mitochondria by oxidative phosphorylation. While 60%–90% of the energy for mitochondrial oxidative phosphorylation is derived from the FAO, the remainder originates from oxidation of glucose, lactate, etc. [34]. RV hypertrophy leads to decreased mitochondrial respiration and disruption of fatty acid metabolism, consequently leading to increased glycolysis. Increased glycolysis in the RV was documented by much greater uptake of 18F-fluorodeoxyglucose in PAH and is associated with higher PVR and RV systolic pressure (RVSP) [53].

**Figure 4:**
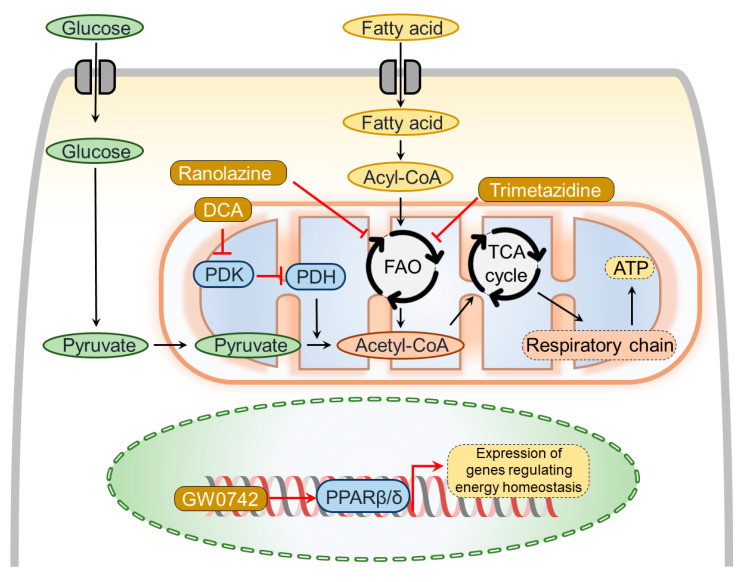
Mechanisms of agents improving metabolic alterations in pulmonary artery banding (PAB)-induced right ventricular (RV) remodeling. Dichloroacetate (DCA) inhibits pyruvate dehydrogenase kinase (PDK), resulting in activation of pyruvate dehydrogenase (PDH), which allows pyruvate to enter the TCA cycle and promote glucose oxidation. While trimetazidine and ranolazine inhibit fatty acid oxidation (FAO), resulting in activation of glucose oxidation, GW0742 activates PPARβ/δ, which mediates the expression of genes regulating metabolic homeostasis. Cumulatively, these agents showed beneficial effects on the RV by improving metabolic changes.

Further dysregulation of metabolic homeostasis is tightly linked to the transition of RV hypertrophy to RV failure [54,55]. For example, congestive heart failure development is accompanied by reduced mitochondrial oxidative phosphorylation and reduced ATP production [54]. As a consequence of decreased mitochondrial oxidative phosphorylation, glucose uptake and glycolysis are elevated in the failing heart [55]. Despite increased glucose uptake, mitochondrial glucose oxidation remains impaired, resulting in an uncoupling of glycolysis from glucose oxidation. The shift from glucose oxidation towards glycolysis is orchestrated by the activation of pyruvate dehydrogenase kinase (PDK) that phosphorylates and inhibits pyruvate dehydrogenase (PDH) [56]. PDH is a key enzyme that converts pyruvate to acetyl-CoA in mitochondria. The accumulation of hypoxia-inducible factor 1 could induce the activation of PDK1 [57] (Figure 4).

Furthermore, RV failure promotes the alteration in fatty acid metabolism. Since FAO consumes 12% more oxygen per mole of ATP generated than does glucose oxidation, inhibiting FAO to increase glucose oxidation is expected to generate more ATP molecules to a given oxygen level, especially in the ischemic myocardium [58]. Moreover, the PH-induced abnormalities in fatty acid metabolism evoke lipid accumulation in the form of triglycerides and diacylglycerols that further promote RV dysfunction [34]. Thus, modulating cardiac energy metabolism by inhibiting FAO and thereby increasing glucose oxidation represents a promising therapeutic approach for both LV and RV failure. Recently performed experimental studies in PAB models have shown that drugs modulating metabolic dysregulation improved RV adaptation and function in response to fixed pressure overload [59,60] (Figure 4).

### 5.1. Peroxisome Proliferator-Activated Receptor Agonists

Peroxisome proliferator-activated receptors (PPARs) are members of the ligand-activated nuclear hormone receptor family [61]. There are three PPAR isoforms, PPARα, PPARβ/δ, and PPARγ, which heterodimerize with the retinoid X receptor (RXR) and bind a PPAR response element (PPRE) to activate/inhibit the expression of a vast set of target genes. PPARs regulate expression of genes involved in maintaining energy homeostasis [61]. A variety of natural and synthetic agents including fatty acids, eicosanoids, and arachidonic acid derivatives can serve as activators of the PPARs. However, the true endogenous PPAR ligands have not been identified yet. Studies have shown that the myocardial expression of PPARβ/δ is higher, compared to other PPARs, and consequently PPARβ/δ is the main PPAR regulating the expression of genes involved in cardiac FAO [62]. Mice with cardiomyocyte-specific deletion of PPARβ/δ display severe impairments in myocardial FAO gene expression, diminished rates of FAO, increased cardiac lipid accumulation, and lipotoxicity with subsequent development of cardiomyopathy [63]. In contrast, cardiomyocyte-specific PPARβ/δ activation prevents adverse LV remodeling in response to pressure overload [64].

Cumulatively, these studies suggest that the presence of functional PPARβ/δ is crucial to maintain LV function and alleviate pressure overload-induced LV remodeling. Similarly, PPARβ/δ has been studied in RV hypertrophy and failure models. For example, testing the PPARβ/δ agonist GW0742 in the rat model of PH induced by chronic hypoxia revealed that GW0742 attenuates pulmonary hypoxic pulmonary vasoconstriction and improves RV function without effecting pulmonary vascular remodeling [65]. The administration of PPARβ/δ agonist GW0742 in PAB mice with established RV remodeling improves RV function and attenuates RV fibrosis and remodeling [66]. Taken together, PPARβ/δ exerts beneficial influence on the RV without affecting the pulmonary vasculature. However, the therapeutic benefits of other PPAR agonists targeting either PPARα or PPARγ on RV adaptation in response to fixed pressure overload remain to be studied (Figure 4).

### 5.2. Agents Modifying Fatty Acid Metabolism

Trimetazidine and ranolazine inhibit FAO, and thereby increase glucose oxidation. Trimetazidine has been shown to attenuate ischemia-induced cardiac dysfunction by inhibiting FAO in both experimental [67,68] and clinical studies [69]. Importantly, trimetazidine increases glucose oxidation both during and after myocardial ischemia [67,68], which attenuates the severity of pH changes in cardiomyocytes in response to ischemia and improves contractile function. This metabolic action can explain the beneficial effects of trimetazidine in the clinical setting of myocardial ischemia [70,71]. Similarly, ranolazine also has been shown to improve ischemia reperfusion-induced cardiac dysfunction [72]. However, inhibitory effects of ranolazine on fatty acid metabolism require high doses, as it primarily inhibits sodium currents [73]. Along these lines, both trimetazidine and ranolazine have been shown to improve metabolic, molecular, electrocardiographic, functional, and hemodynamic characteristics of the RV in response to fixed pressure overload [59]. Importantly, both drugs have been able to not only prevent but also to reverse established RV remodeling and to improve RV dysfunction in PAB rats [59].

Recently, emerging evidence demonstrated beneficial effects of sodium-glucose co-transporter-2 (SGLT2) inhibitors in the treatment of heart failure with reduced ejection fraction (HFrEF) [74]. SGLT2 inhibitors, besides inhibiting a glucose reabsorption in the proximal tubule of the kidney, affect lipid metabolism by decreasing lipid accumulation, reducing lipid oxidation, and shifting substrate utilization towards the usage of ketone bodies [75]. Moreover, the SGLT2 inhibitor empagliflozin reduced mortality and prevented progression of MCT-induced PH [76]. However, effects of SGLT2 inhibitors on RV failure in PAB model have not been investigated yet.

Taken together, inhibition of FAO may be beneficial to improve RV adaptation and prevent maladaptive remodeling in response to fixed pressure overload (Figure 4).

### 5.3. Agents Restoring Oxidative Phosphorylation

During physiological conditions, PDH converts pyruvate to acetyl CoA, which in turn is utilized as a substrate in the tricarboxylic acid (TCA) cycle. However, with the development of RV failure, mitochondrial oxidative phosphorylation is impaired partly due to upregulation of PDK, which inhibits PDH [77]. This change can be therapeutically targeted by dichloroacetate (DCA), which inhibits PDK activity. DCA has been shown to improve RV function in both MCT and PAB rats [60]. Importantly, DCA affects beneficially both the pulmonary vasculature and the RV. However, the beneficial effect of PDK inhibition on RV is afterload-independent, since DCA improves RV performance even in a PAB model with fixed RV pressure overload, although the benefits of DCA on the RV are greater in PH models than in the PAB model due to exerting beneficial effects on both the pulmonary vasculature and the RV [59,60]. Interestingly, DCA treatment improves cardiac electromechanical remodeling by normalizing QTc (corrected QT interval) in MCT rats [60]. In addition, in a four-month phase I trial in patients with idiopathic PAH, who already had approved PAH therapy, DCA treatment (3 to 6.25 mg/kg b.i.d.) led to overall improvement of hemodynamics and functional capacity with a high degree of interindividual differences [78]. PAH patients with a poor response to DCA carried functional inactivating variants of genes encoding sirtuin-3 (SIRT3) and uncoupling protein 2 (UCP2) [78], suggesting that the presence of physiologically functioning SIRT3 and UCP2 is crucial for the manifestation of DCA benefits. In conclusion, DCA may support RV adaptation in response to the pressure overload by restoring cardiomyocyte glucose oxidation (Figure 4).

## 6. Pharmacotherapies Modulating Myocardial Inflammation and ROS

**Figure 5:**
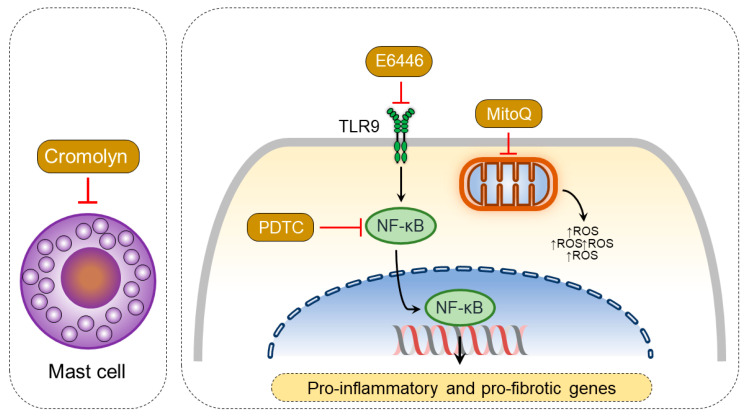
Mechanisms of agents attenuating myocardial inflammation and oxidative stress in pulmonary artery banding (PAB)-induced right ventricular (RV) remodeling. E6446, an inhibitor of TLR9, and pyrrolidine dithiocarbamate (PDTC), an inhibitor of NF-κB, attenuate inflammatory cell activation and expression of proinflammatory genes. Cromolyn, a mast cell stabilizer, inhibits mast cell degranulation and prevents the release of proinflammatory mediators. MitoQ inhibits mitochondria-derived ROS production and oxidative stress. In summary, these agents inhibit myocardial inflammation and oxidative stress and thereby improve RV function and adaptation to pressure overload.

Cardiovascular diseases are generally associated with upregulation of inflammation, which is suggested as one of the main drivers of pathological processes, ultimately serving as a potential therapeutic target [79]. However, there are no approved anti-inflammatory therapies for cardiovascular diseases so far. Various proinflammatory cytokines and chemokines are released during pressure overload induced by myocardial injury, which can promote hypertrophic and profibrotic responses [80]. In experimental models of LV hypertrophy, anti-inflammatory strategies have been shown to improve LV remodeling and function in response to pressure overload [81]. Similarly, many chemokines and cytokines are dysregulated in the pressure-overloaded RV [25,82]. Moreover, some of them have been targeted to improve RV function in PAB models. For example, an increased content of mast cells was observed in the RV upon pressure overload in PAB mice [83]. The mast cell stabilizer cromolyn prevented and reversed RV remodeling in PAB mice when applied from day 1 or day 7 after PAB operation, respectively [84]. Furthermore, both NF-κβ and TLR9 inhibitors could improve RV remodeling, fibrosis, and function by attenuating myocardial inflammation in PAB rats [85]. In addition, rapamycin could attenuate RV hypertrophy due to immunosuppressant effects [86]. In summary, attenuating RV myocardial inflammation may serve as an important strategy in the treatment of RV failure in response to pressure overload (Figure 5).

ROS are key signaling molecules underlying various inflammatory responses [87]. In addition to inflammatory cells, other cardiac cells are also involved in increased oxidative stress in numerous cardiac pathologies [88]. Different conditions such as myocardial ischemia, cardiomyopathies, as well as pressure and volume overload induce oxidative stress in the myocardium of both ventricles. For example, a sustained increase of afterload causes an increase of mitochondrial [33,89] and non-mitochondrial ROS [89,90] production in the RV. Downstream signaling pathways underlying ROS-mediated alterations in development of RV failure are not completely understood. However, available studies suggest that altered ROS production could modify the structure/function of various proteins, including matrix metalloproteases [91] and p38 MAP kinase [89], and thus contribute to adverse myocardial remodeling. Recently, it has been demonstrated that inhibition of mitochondrial ROS release by mitochondria-targeted MitoQ, which is a derivative of coenzyme Q, attenuated RV dysfunction and remodeling without effects on pulmonary vascular remodeling in mice exposed to 10% O_2_ chronic hypoxia for 4 weeks. This suggests a differential role of mitochondrial ROS in the RV vs. the pulmonary vasculature. Importantly, the RV-specific benefit of MitoQ, such as attenuated RV dilatation and improved RV function, was maintained even in a mouse PAB model, suggesting RV-directed effects of MitoQ. Summarily, inhibition of myocardial oxidative stress is one of the approaches to improve RV function and adaptation to pressure overload (Figure 5).

## 7. Pharmacotherapies Modulating Myocardial Epigenetics

Alterations of various epigenetic mechanisms such as DNA methylation, histone modification, chromatin remodeling, and noncoding RNAs have been shown to contribute to various cardiovascular diseases, including LV hypertrophy and failure [92]. Moreover, pharmacological agents that target altered epigenetic mechanisms in various preclinical models of LV failure have been shown to improve cardiac function [93].

Among noncoding RNAs, long noncoding RNAs (lncRNAs) have emerged as important epigenetic regulators to control chromatin function and gene expression. LncRNAs are noncoding RNA transcripts consisting of 200 or more nucleotides with biochemical properties to interact with diverse groups of molecules such as RNA, DNA, and proteins, subsequently regulating their functions [94]. Dysregulation of diverse lncRNAs has been shown to contribute to LV hypertrophy and failure in response to pressure overload [95]. Contrary to LV failure, the roles of lncRNAs in RV failure have not been studied extensively. However, few lncRNAs have been identified to play a role in RV hypertrophy and failure. For example, a recent study revealed that lncRNA H19 is upregulated in decompensatory RV remodeling compared to compensated RV hypertrophy in PAH patients and normal RV of healthy controls [96]. Similarly, H19 was upregulated in the decompensated RV in MCT-induced as well as in PAB models. Interestingly, H19 upregulation was specific to the RV in both rats and humans as no change was observed in either the LV or lungs of humans and rats, even with decompensated RV remodeling [96]. Importantly, silencing H19 expression reduced cardiomyocyte hypertrophy and myocardial fibrosis, improved myocardial capillary rarefaction, and preserved RV function without affecting pulmonary vascular remodeling [96]. The therapeutic effects of H19 antagonism are caused by the upregulation of the histone methyltransferase enhancer of zeste homolog 2 expression [96]. Interestingly, circulating levels of H19 indicate disease severity and prognosis of PAH patients [96].

Posttranslational modifications of core histone proteins regulate chromatin architecture in a condensed (inactive) versus open (active) state. These processes tightly regulate chromatin structure, eventually modulating cellular transcriptional responses [93]. Reversible incorporation of acetyl groups within histone tails is one of the best studied epigenetic processes regulating chromatin structure and gene expression. Histone deacetylases and their inhibition have been extensively studied and characterized in various models of LV diseases [97]. Thus, initial studies using rodent models of LV failure revealed that HDAC inhibition mitigates pressure overload-induced LV hypertrophy and failure [98,99]. However, in contrast to the beneficial effect of HDAC inhibitors in LV failure, the HDAC inhibitors trichostatin A and valproic acid even further deteriorated RV function when applied after established RV remodeling in PAB rats. This effect was mainly due to exaggerated myocardial fibrosis and capillary rarefaction [100]. However, in another study, valproic acid, applied in a preventive approach, attenuated RV remodeling and maintained RV function in PAB rats, as evidenced by attenuated RV hypertrophy fibrosis and improved RV function [101].

**Figure 6:**
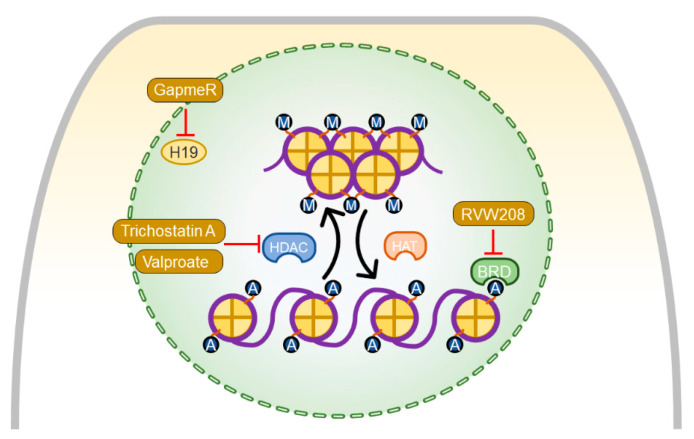
Mechanisms of agents improving epigenetic alterations in pulmonary artery banding (PAB)-induced right ventricular (RV) remodeling. Trichostatin A and valproate target histone deacetylases (HDACs), which remove acetyl groups from lysine residues of histone proteins. RVW208 is an inhibitor of bromodomain-containing protein 4 (BRD4), which is an epigenetic reader that recognizes histone proteins and acts as a transcriptional regulator of various genes. GapmeR, a site-specific antisense oligonucleotide, suppresses the expression of long noncoding RNA H19. All these agents mediate the improvement of RV function and promote its adaptation to pressure overload.

Another group of regulators of epigenetic mechanisms, bromodomain and extraterminal domain (BET) family proteins, has recently become of great interest as promising treatment targets in diverse cardiovascular diseases [102]. The BET family proteins, including BRD2, BRD3, BRD4, and BRDT, are epigenetic readers that bind acetylated histone tails via protein interaction domains, facilitating the localization of transcription factors and other coactivators to regulate a vast network of transcriptional programming [103]. Inhibitors of BET family proteins have been shown to improve cardiac function in LV failure models. For example, the pan-BET inhibitor JQ1 has been shown to be effective in attenuating LV remodeling in a mouse transverse aortic constriction (TAC) model [104]. Based on the observation of the increased expression of BRD4 in the coronary vasculature of the remodeled RV in PAH patients and MCT rats [105], inhibition of BRD4 by apabetalone (RVX-208) in PAB rats was found to improve adaptation to pressure overload. Interestingly, apabetalone also exerted profound effects on pulmonary vascular remodeling in MCT-induced and SuHx rat models of PH [106]. In summary, modulating epigenetic changes seem to improve RV adaptation to pressure overload (Figure 6).

## 8. Pharmacotherapies Modulating Myocardial Regenerative Mechanisms

Tissue regeneration, tissue engineering, and repair using stem cells are the most promising methods to develop biological therapeutic strategies in various diseases. Stem cells can be generated from both embryonic and adult tissues, which are characterized by their ability to proliferate and produce mature functional cells. In addition, autologous patient-derived cells can be used with or without ex vivo modifications. In heart failure, stem cell therapy is expected to at least partially restore the functional capacity of injured cardiac tissue. Stem cells can either be cultured and expanded ex vivo, before they are transplanted to functionally integrate into the cardiac myocardium, or they can be used for ex vivo tissue engineering using complex 3D models.

Initially, it was claimed that cardiac functional improvement following stem cell therapy in preclinical models of heart failure is due to differentiation of stem cells into functional cardiomyocytes. However, later studies using lineage tracing have been unable to show true (trans-) differentiation into cardiomyocytes, although overall benefits to the cardiac function remained [107]. Thus, the hypothesis of paracrine effects of stem cells on cardiac function has been pursued by many researchers [108]. Most recently, a study has revealed that the functional benefit of stem cell therapy following myocardial infarction is due to an acute inflammatory-mediated wound healing response that rejuvenates the mechanical properties of the infarcted area of the LV [109]. Thus, current knowledge suggests that therapeutic effects of stem cell therapy in heart failure are mediated by modification and promotion of inflammation-induced endogenous healing processes.

Various sources of stem cells, including bone marrow-derived mononuclear cells (BM-MNCs), umbilical cord blood-derived mononuclear cells (UCB-MNCs), and cardiosphere-derived cardiac progenitor cells (CDCs), have been investigated for stem cell therapies in heart failure. Both BM-MNCs and UCB-MNCs have been shown to be suitable for intracoronary injection as well as for intramyocardial transplantation, although these cells (c-kit+) exhibit only minimal transdifferentiation potential into cardiomyocytes [110]. CDCs represent the most promising cell type for cardiac regeneration due to their myocardial tissue origin. CDCs can be extracted, isolated, and expanded from the myocardial tissue of patients and can then be administered by intramyocardial injection or intracoronary infusion [111]. CDCs were shown to promote regeneration of cardiac tissue through the recruitment of progenitor cells and promotion of resident cardiomyocyte proliferation [112]. In animal models of heart failure, CDCs were demonstrated to exert regenerative, cardioprotective, anti-inflammatory, and antifibrotic effects [113]. However, large-scale clinical trials in heart failure patients showed that stem cells do not improve cardiac function and survival [114].

In the PAB model of RV hypertrophy and failure, various stem cell types have been studied, including BM-MNCs, UCB-MNCs, and CDCs [115]. For example, administration of neonatal BM-MNCs in a mouse PAB model attenuated RV hypertrophy and dilatation [115]. In addition, intramyocardial delivery of human BM-MNCs in a neonatal pig PAB model improved myocardial capillarization, increased the number of c-kit+ stem cells, and increased endothelial cell proliferation, eventually resulting in reduced RV hypertrophy and improved RV function [116]. In PAB mice, human UCB-MNCs reduced myocardial fibrosis and increased myocardial capillarization [117], and in an ovine PAB model, transplantation of human UCB-MNCs improved RV mechanical properties such as RV compliance and recruitable stroke work [118]. Similarly, intramyocardial injection of c-kit+ cells prevented RV dilatation, reduced RV fibrosis, and improved RV function in PAB piglets [119]. Mechanistically, human CDC-derived exosomes in a porcine PAB model have attenuated RV hypertrophy, myocardial capillarization, and RV function [120].

Taken together, currently available studies suggest that stem cell-based therapies act beneficially on the RV in response to fixed pressure overload largely due to improvements in myocardial angiogenesis, reduced cardiomyocyte hypertrophy, and decreased fibrosis, thus improving RV performance.

## 9. Conclusions

**Figure 7:**
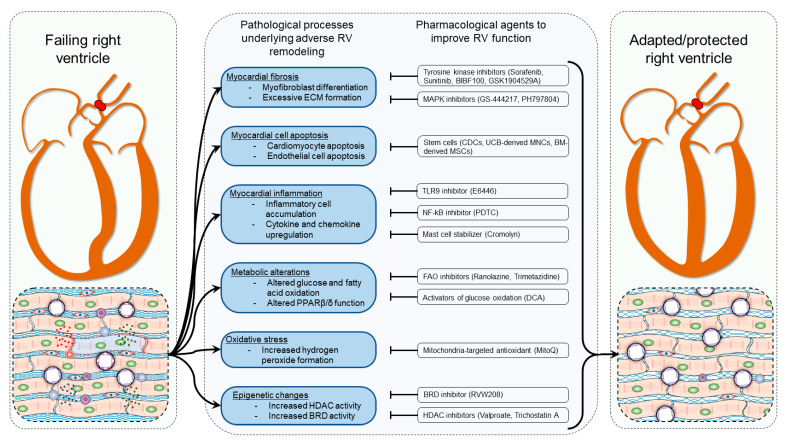
Main pathological features of right ventricular (RV) remodeling and main signaling pathways targeted to improve RV adaptation and protection in response to pressure overload. Upon exposure to pressure overload, the RV undergoes changes such as RV wall hypertrophy and dilatation, associated with various pathological processes including myocardial fibrosis, apoptosis of myocardial cells, myocardial inflammation, oxidative stress, metabolic remodeling, and alterations of epigenetic processes. Targeting the signaling pathways driving such changes improves RV function and adaptation to pressure overload.

Right heart failure is a big challenge for development of successful therapeutics in PAH. The current knowledge derived from the PAB model of RV hypertrophy and failure in animals suggests that various pathological conditions such as myocardial fibrosis, cellular apoptosis, myocardial inflammation, metabolic alterations, oxidative stress, and epigenetic changes contribute to adverse RV remodeling. Targeting the signaling pathways driving such changes seems to increase RV adaptation and promote RV protection in response to pressure overload (Figure 7). Thus, use of the PAB model has led not only to the discovery of potential therapeutic targets but also allowed to study RV-directed drugs. However, only a small selection of experimental drugs with documented benefits for the pulmonary vascular remodeling have been tested in PAB models with respect to their direct effect on the heart and respective beneficial or adverse effects in the RV. Importantly, some of the experimental drugs entered clinical trials without proper characterization of their effects on the RV, which may have devastating consequences for PAH patients enrolled for such studies if the drug turns out to have, for example, cardiotoxic effects. Thus, the PAB model should be utilized during development of PAH therapies for the characterization of their safety profile. Proper utilization of the PAB model results in increased knowledge of the pathological mechanisms underlying RV failure, which may subsequently lead to the discovery of new treatment targets. Thus, increased focus of the research on RV physiology and pathology increases the chances of finding a successful therapy for the treatment of RV failure. The results of the above-discussed experimental therapeutic strategies developed using the PAB model are promising. Moreover, despite beneficial effects of the agents discussed above on the RV, further research is required before forwarding those agents to clinical trials to become, hopefully, efficacious therapeutic options.

## Data Availability

Not applicable.

## Abbreviations

α-SMA, actin alpha 2; ACE, angiotensin-converting enzyme; ASK1, apoptosis signal-regulating kinase 1; BET family, bromodomain and extraterminal domain family; BM-MNCs, marrow-derived mononuclear cells; BRD, bromodomain; CDCs, cardiosphere-derived cardiac progenitor cells; CSF1R, colony-stimulating factor 1 receptor; CTEPH, chronic thromboembolic pulmonary hypertension; DCA, dichloroacetate; ECM, extracellular matrix; ERK1/2, extracellular signal-regulated ki-nases; FAO, fatty acid oxidation; FGFRs, fibroblast growth factor receptor; Flt3, fms-related receptor tyrosine kinase 3; HDAC, histone deacetylase; HFrEF, heart failure with reduced ejection fraction; IGF-1R, insulin-like growth factor 1 receptor; JNK, c-jun N-terminal protein kinases; lncRNAs, long noncoding RNAs; LV, left ventricle; LVAD, left ventricular assist device; MAP3K, mitogen-activated protein kinase kinase; MAPKs, mitogen-activated protein kinases; MCT, monocrotaline; MMPs, matrix metalloproteinases; mPAP, mean pulmonary artery pressure; MRTF-A, myocardin-related transcription factor A; mTORC1, mammalian target of rapamycin complex 1; NF-κB, nuclear factor kappa-light-chain-enhancer of activated B cells; PAB, pulmonary artery banding; PAH, pulmonary arterial hypertension; PAP, pulmonary artery pressure; PDGFRs, platelet-derived growth factor receptors; PDH, pyruvate dehydrogenase; PDK, pyruvate dehydrogenase kinase; PDTC, pyrrolidine dithiocarbamate; PH, pulmonary hypertension; PPARs, Peroxisome proliferator-activated receptors; PPRE, PPAR response element; PVR, pulmonary vascular resistance; RAAS, renin–angiotensin–aldosterone system; ROS, reactive oxygen species; RV, right ventricle; RXR, retinoid X receptor; SGLT2, sodium-glucose co-transporter-2; SIRT3, sirtuin-3; SuHx, SU5416 plus chronic hypoxia; TAC, transverse aortic constriction; TCA, tricarboxylic acid; TGF-β, transforming growth factor beta; TIMPs, tissue inhibitors of metalloproteinases; TKIs, tyrosine kinase inhibitors; TLR9, toll-like receptor 9; UCB-MNCs, umbilical cord blood-derived mononuclear cells; UCP2, uncoupling protein 2; VEGFRs, vascular endothelial growth factor receptors.
